# Formulation of Fast Dissolving β-Glucan/Bilberry Juice Films for Packaging Dry Powdered Pharmaceuticals for Diabetes

**DOI:** 10.3390/plants11152040

**Published:** 2022-08-04

**Authors:** Ionut Avramia, Sonia Amariei

**Affiliations:** Faculty of Food Engineering, Stefan cel Mare University of Suceava, 720229 Suceava, Romania

**Keywords:** fast dissolving films, yeast β-glucan, bilberry juice, diabetes

## Abstract

The aim of this study was to develop fast dissolving films based on β-glucan and bilberry juice due to the bioactive potential of β-glucan and antidiabetic effect of bilberry juice. The benefit of incorporation of bioactive compounds into the films is due to the removal of unnecessary excipients and to confer protection as well as increase stability and shelf life to the packaged product. Due to the fast dissolving requirements of the European Pharmacopeia, which reduced the dissolution time from 180 to 60 s, indicating less than a minute, hygroscopic materials, such as sodium alginate and a suitable plasticizer, such as glycerin were incorporated. Moreover, the influence of ingredients and surfactants, such as soybean oil was studied in the design of fast dissolving films. Additionally, the steady state rate water vapor transmission rate (WVTR), water vapor permeability (WVP), and FT-IR spectroscopy tests were performed at high resolution to ensure the reliability of the films and composition as well as to validate the results. Our data suggest that the addition of surfactants contributed to the development of fast dissolving films without influencing the diffusion of water vapor. Low levels of WVTR and short dissolution time made from β-glucan and bilberry juice are a convenient candidate for packaging dry powdered pharmaceuticals for diabetes.

## 1. Introduction

Films based on different bioactive compounds are defined as a new generation of edible films due to the active agents, which act as antimicrobials, anti-inflammatory, antioxidants, immunostimulants, anti-cancer, etc. [1,2,3]. There are plenty of natural compounds that can be incorporated into the film-forming solution for film making, depending on their role in therapeutics and the related medical condition. Films with strong antioxidant properties were successfully developed using natural compounds, such as plants from *Moringaceae* family [4], chitosan/ellagic acid films were found to have strong antimicrobial activity against *S. aureus* and *P. aeruoginosa* [5], while β-glucan/pomegranate juice films had potential in the management of diabetes through the variety of tannins and bioactivities [6].

Diabetes is the most common noncontagious disease in the world [7]. A study conducted in 2019 estimates that this serious, long-term condition with a major impact on the lives and well-being of individuals around the world affects about 463 million people. It was estimated that, by 2030 this number will increase to 578 million people and in 2045 it will increase to 700 million people (equivalent to 10.9% of the population), with over 200 million more than 2019 [8].

Functional ingredients, such as bilberry (*Vaccinium myrtillus* L.) are recognized since centuries to have antidiabetic potential and have been used to control blood sugar levels [9]. A comprehensive review in 2022 of the phytochemical and pharmacological antidiabetic properties of bilberries by Chehri et al. (2022) highlighted the beneficial properties of the most significant components from the fruit. In addition to the antidiabetic effects, the authors analyzed the cardioprotective effects, anti-obesity, anti-inflammatory or ocular disorder effects, with all of these found in diabetes-related complications [10].

Yeast β-glucan is a complex polysaccharide of β-1,3, which is linked with β-1,6 glucose polymers that are found in the cell wall of yeast [11]. The biological effects of the β-glucans are related to the immunity, anti-cancer, and anti-inflammatory properties of the body with a mechanism of action not yet fully understood [12]. It is known to act by binding to Pattern Recognition Receptors, such as Dectin-1, LacCer, CR3, and SR3 [13]. By stimulating the Dectin-1 signaling pathway, β-glucans might confer protection of β pancreatic cells against the T cells in T1D [14]. Several authors have studied the oral dispersible polysaccharides film with pullulan (a linear structure of glucan) as a drug delivery system for treatment of diabetes [15], while other researchers used immediate-release layers of coatings to prepare fixed-dose combination tablets for diabetes [16].

Taken together, a fast dissolving film that allows the incorporation of these bioactive compounds is the aim of this study. The composition of film-forming solution (FFS) has been chosen to examine the rapid dissolving forms of films. Therefore, the addition of hygroscopic materials, plasticizers, and surfactants was evaluated to observe significant changes between samples. Moreover, for an adequate packaging film, additional tests including thickness, WVTR, WVP, film opacity, water activity, moisture content, and color profile were performed. Finally, further statistical correlation between dependent and independent variables, such as chemical film composition was determined.

## 2. Results

### 2.1. Optimization of the Film Composition Considering Dietary Intake and Total Solids

In order to find an optimal composition for a fast dissolving film with a high content of bioactive compounds, seven samples with different amounts of β-glucan and bilberry juice were cast onto a plastic petri dish with or without soybean oil as surfactant. This allows for the identification of any changes in the surface tension reduction as well as improvements on wettability and adhesion of the film. While the bilberry juice rich in anthocyanins, quercetin derivatives, proanthocyanidins or chlorogenic acid phytochemicals has no dietary intake recommendation, β-glucans are limited by the European Food Safety Authority up to 1.275 g/day of dietary use for the general adult population [17].

From the data presented in Table 1, it can be seen that an equal amount of sodium alginate was introduced in all seven samples, which in relation to the total volume has a concentration of 0.53 % (*w*/*v*). This falls within the limits of 0.125–1.5% and exhibits a pseudoplastic shear flow behavior [18]. Additionally, film sizing was performed to a corresponding area density between 7.82 and 12.19 mg × cm^−2^. The plasticizer (glycerin) with the best compatibility related to β-glucan and most popular for anthocyanin-based films [19,20] was added to the all polymer samples, while the soybean oil was included in three different samples as 2% (*w*/*w*) of the total solids (β-glucan, sodium alginate, and bilberry juice).

### 2.2. Film Thickness

The film thickness expressed in µm is an important characteristic in packaging materials. Different thicknesses are essential to the other properties, such as water barrier, transparency or color attributes [21,22]. The film thickness of β-glucan/bilberry juice films ranges between 66.43 and 119.7 µm and was significant (*p* < 0.001) depending on the total solids in the film-forming solution (2.95 and 4.6 g, respectively). This behavior is in accordance with the observations of Arham et al. (2016) who observed an interaction between film thickness and based materials [23]. Similar results have been obtained by Peltzer et al. (2018) and Zhao et al. (2022), in which thicknesses up to 200 µm contributed to the stability and uniformity of the film making suitable for packaging applications [24,25].

### 2.3. Water Vapor Transmission Rate (WVTR)

WVTR of the β-glucan/bilberry juice films generally has low values between 3.2562 and 7.1111 g × h^−1^ × m^−2^ (Table 2). It was observed that the increasing trend of WVTR was determined by the increase in the total content of substances introduced in the film-forming solution. Additionally, studies conducted by Rahmawati et al. (2020) showed that the amount of plasticizer strongly influenced the water absorption rates [26]. Due to the hydrophilic nature of glycerin, which has three hydroxyl groups, the influence of the plasticizer on the water vapor that permeates the film was investigated. The results are shown in Table 3.

Indeed, WVTR has a tendency to increase with the glycerin content. The maximum value of 7.1111 g × h^−1^ × m^−2^ (Table 2) has the highest value of glycerin content of 1.15 g in the film-forming solution. Interestingly, Sample 7 with the same amount of glycerin has a low value of WVTR of 5.9036 g × h^−1^ × m^−2^. One of the reasons for the difference is due to the fact that in the sample with the highest WVTR value the film does not contain soybean oil, which is known to have hydrophobic nature. Therefore, by decreasing the intermolecular interaction, the mobility of the molecule promotes the migration of water vapor through membranes. Compared with other bilberry-based films, which have values between 52.91 and 61.87 g × h^−1^ × m^−2^ depending on the bilberry concentration [27], WVTR showed low values with a minimum of 3.2562 g × h^−1^ × m^−2^. The addition of β-glucan or pullulan to the films has also been shown to increase moisture barrier properties [28]. Ultimately, of course, our data are 32 higher than the value of pure low density polyethylene (LDPE) film values of 0.1012 g × h^−1^ × m^−2^ found by Reesha et al. (2015) [29]. To date, a bioactive film with potential packaging product applications showed the best barrier properties.

### 2.4. Water Vapor Permeability (WVP)

The results of the water vapor flux through the film (WVP) determined by dividing WVTR value to the differential water vapor partial pressure across the film and multiplied by the thickness of the film (in mm) are presented in Table 2. It was observed that WVP values ranged between 0.1057 and 0.3568 g × mm × kPa^−1^ × h^−1^ × m^−2^, particularly with the increase in bilberry juice and β-glucan content. An increased value of the WVP parameter indicates that the film is more susceptible to water vapor flux penetration [24]. With an investigation of the physicochemical properties of films, and with the knowledge that high polar polymers and the addition of plasticizers resulted in an increased WVP values, Henrique et al. (2007) concluded that vapor permeability can be related to the quantity of –OH groups in the molecule [30]. On the other hand, Garcia et al. (1999) mentioned that coatings without plasticizing agents led to significantly (*p* < 0.05) higher values of WVP than those with plasticizer due to the formation of pores and cracks [31]. Undoubtedly, we can conclude that our data are lower than those found in films made only from β-glucan without the addition of plasticizer. This is the case of research conducted by Sarossy et al. (2013), which reported WVP values of 0.4625 g × mm × kPa^−1^ × h^−1^ × m^−2^ (or 11.1 g × mm × kPa^−1^ × m^−2^ × d^−1^) [32] or by those determined by Peltzer et al. (2018) with an amount of 2.8 × 10^−10^ g × s^−1^ × m^−1^ × Pa^−1^ [24].

### 2.5. Dissolution Time

One of the most important aspects in developing a fast dissolving film is the time that should not exceed 1 min [33]. Significant differences between samples (*p* < 0.001) are observed in Table 2 with a higher variation between sample means relative to the variation within the samples (an F-value of 674.08). The values of the dissolution time range from 22.33 to 105.66 s. This is mainly due to the composition of the film-forming solution. While soybean oil behaves as a good surfactant [34], Rodriguez et al. (2006) investigated the combined effect of plasticizers and surfactants on the physical properties of films. The authors concluded that surfactants improved the wettability properties of the film solutions by decreasing the surface tension and in combination with glycerin allowed a higher molecular mobility [35]. To verify whether the composition significantly influenced the dissolution time, Table 4 investigated the film composition and the addition of surfactant on dissolution time.

In Table 4, we can observe that the incorporation of β-glucan between 1 and 1.5 g in films does not have a significant influence on dissolution time (*p* > 0.05; *p* = 0.3). On the other hand, data analysis showed that soybean oil and bilberry juice content have significant influence on the dissolution time of the films (*p* < 0.05 and *p* < 0.001, respectively). The obtained F-value of 20.06, which is significant at 0.1%, indicates that there is a significant influence of bilberry juice on dissolution time. High content in the amount of bilberry juice had a negative effect in terms of dissolution time of the films with an average of 74.08 s at 20 g compared with 31.44 s at 10 g of added bilberry juice. At the same time, the addition of 2% soybean oil to the film-forming solution showed a significant decrease (5% level of significance) in the dissolution time.

By correlation, for example, we can observe that in Table 1 the composition of Sample 7 differs from Sample 6 only by the addition of soybean oil, with other compounds remaining in the same proportion. On the other hand, in Table 2, we can observe a major difference in the dissolution time of 50.33 s in the sample with the addition of surfactant and 105.66 s without the soybean oil. Finally, we can summarize that the addition of surfactant positively influences the dissolution time and acts as a solubilizing agent.

### 2.6. Water Activity Tests (a_w_) and Moisture Content (MC)

While the statistical analysis of the water activity tests showed significant differences between samples (*p* < 0.001), the data indicate narrow values between 0.3562 and 0.3915. However, our results are below the critical values where microbiological spoilage can occur. Majumdar et al. (2018) summarized in a review that a minimum water activity value of 0.6 is required to initiate the growth of microorganisms [36]. These data are also consistent with more in-depth research by Beuchat (1983), which concluded that below 0.61 a_w_ there can be no microbial growth, between 0.61 and 0.85 a_w_ food spoilage starts with mold and yeast formation, and above 0.85 a_w_ bacteria start to grow [37].

Another important parameter in food packaging applications is that the moisture content varied between 10.19 and 14.39%. Moisture content of films is closely related to the total amount of water molecules in the network microstructure of the composite films [38]. Abdalrazeq et al. (2019) stated that a high MC considerably limits the use of coatings for packaging materials and found the highest moisture value of 33.27% in film samples with 50% of glycerin prepared at pH 7 [39].

### 2.7. Color and Opacity

Consumer acceptance is influenced by two parameters of the film appearance: Color and opacity. The opacity and L, a*, b* values presented in Table 2 were significantly different. The two main factors that varied in the film-forming solution, β-glucan and bilberry juice, have been evaluated in Table 5 for their influence on the opacity, brightness characteristics of L value (between 0 and 100), redness/greenness (a* value), and yellowness/blueness (b* value).

The results in Table 5 showed that a high amount of β-glucan is reflected by an increased brightness, a tendency to blueness, and an opaquer film, while the a* parameter does not show a significant variation (*p* > 0.05). Regarding bilberry juice, a significant variation (*p* < 0.001) can be observed between the addition of 10 and 20 g to the red color of the samples (a* value). L and b* parameters for bilberry juice are not statistically significant (*p* > 0.05), while the opacity increased significantly (*p* < 0.05).

### 2.8. Scanning Electron Microscopy (SEM)

**Figure 1:**
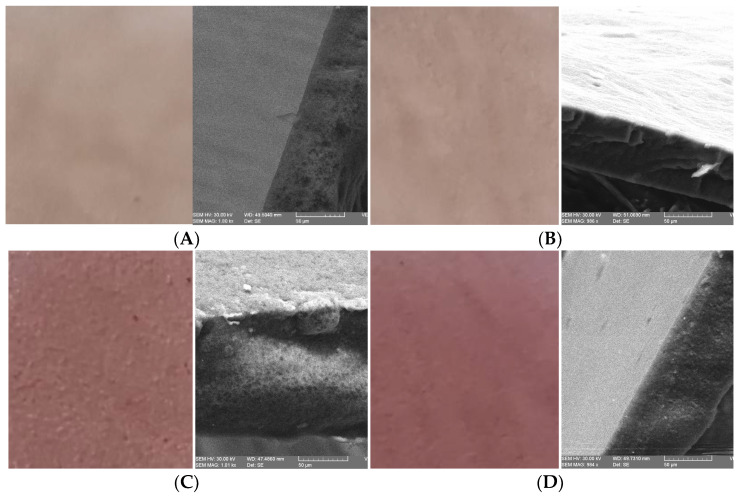

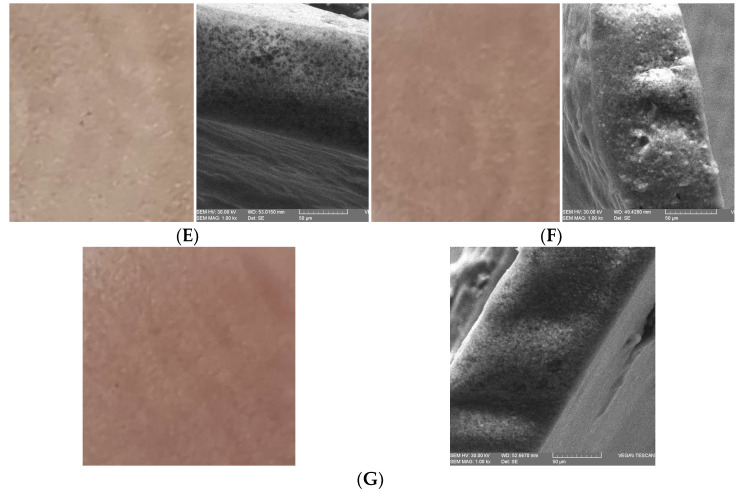
β-glucan/bilberry juice images and SEM micrographs of the film samples. (**A**–**G**) indicate the film samples from 1–7.

Figure 1 shows the microstructure of the β-glucan/bilberry juice films in the cross section at 1 kx to analyze the differences between surfactant and non-surfactant samples and to observe potential microcracks. As can be seen, on the transverse section, the samples with 2% surfactant (Figure 1B,D,G) showed a more compact surface, while the remaining samples present porous surfaces with micropores. The presence of pores makes the film less efficient in the water vapor barrier performance.

### 2.9. FT-IR Spectroscopy

**Figure 2:**
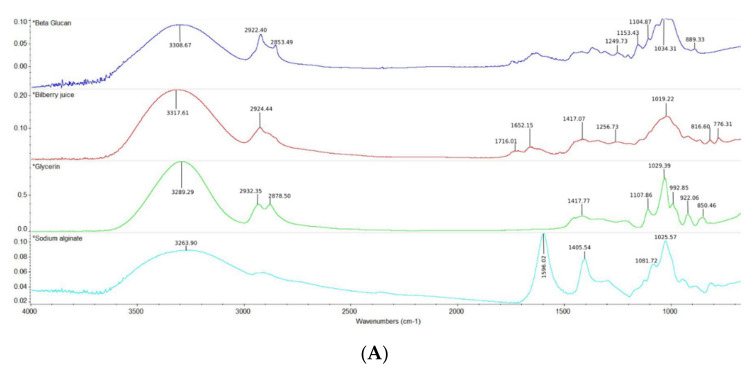

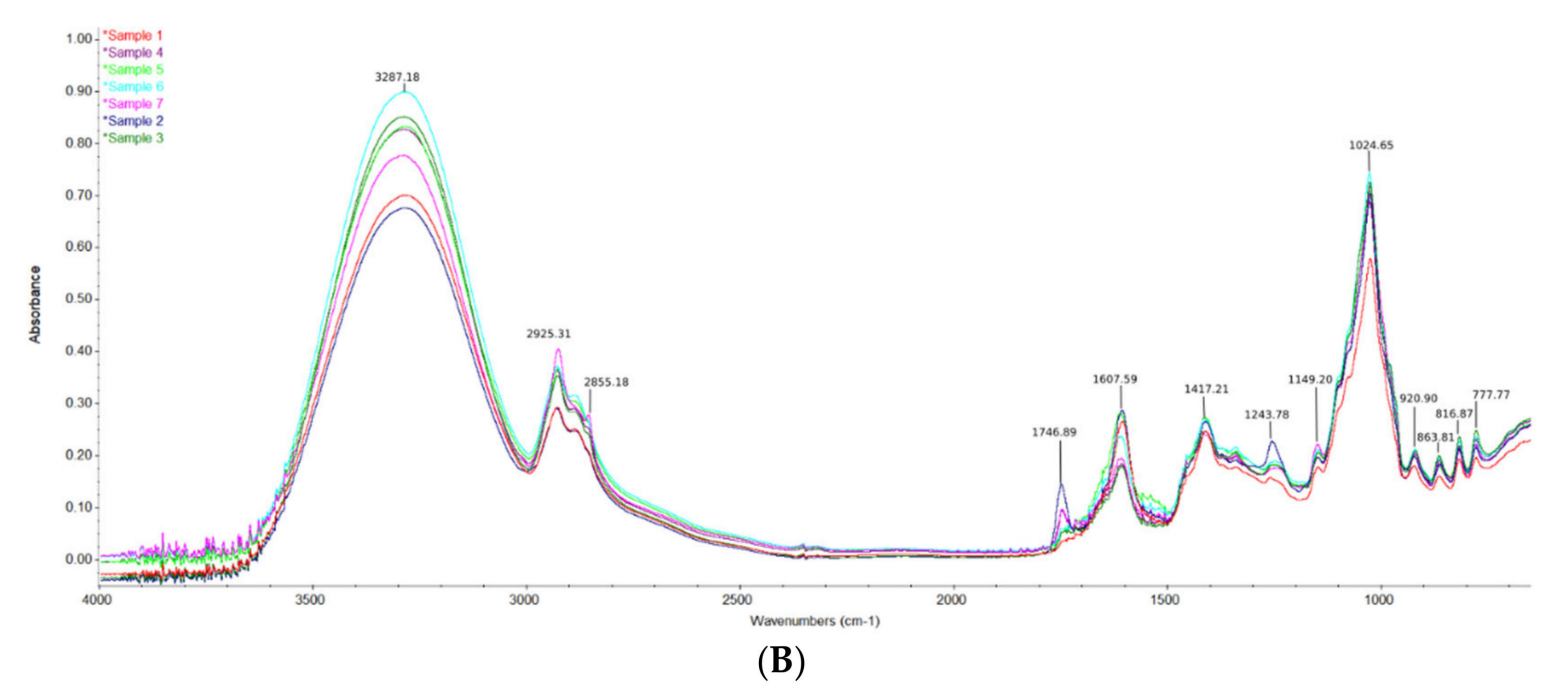
ATR−FTIR spectra: (**A**) Individual FT−IR spectra for pure samples of BG, BJ, GLY, SA; (**B**) FT−IR spectra of the β-glucan/bilberry juice film samples (4000−650 cm^−1^).

The ATR-FTIR spectra (Figure 2A) of the most abundant group of polyphenols from bilberry juice (rich in anthocyanins) are identified by peaks found near the wavenumbers of ∼1716.01 cm^−1^ (C=O stretching for aromatic nucleus) [40], ∼1652.15 cm^−1^ characteristic for benzene skeleton vibration in anthocyanins, while the 3317.61 and 2924.44 cm^−1^ are assigned to O–H stretching vibration of water and CH, CH_2_, and CH_3_ groups, respectively [41], and ∼1417.07 cm^−1^ corresponds to the C–H deformation [42]. Figure 2B showed that the incorporation of bilberry juice in films preserves characteristic peaks in the fingerprint region of the BJ, which indicates that the heat treatment of 15 min from the addition of bilberry juice to obtain the film-forming solution did not affect the bioactive compounds in the obtained β-glucan/bilberry film. Moreover, peaks near the wavenumbers of ∼1149.20, 1024.65, and 920.90 cm^−1^ are characteristic for yeast β-glycosidic configuration, sodium alginate (carboxyl stretching bands), and glycerin, respectively [43,44,45].

## 3. Discussion

In this study, fast dissolving films from β-glucans and bilberry juice were successfully prepared. In addition to the role of plasticizer and sodium alginate, which confers vital primary film structure characteristics for a rapid dissolution, the addition of surfactant significantly reduces the dissolution time by improving the solubility of the β-glucans/bilberry juice films. In the case of fast dissolving films, this becomes very important, particularly when dispersing compounds, such as β-glucans or bioactive compounds from bilberry juice with antidiabetic properties, such as anthocyanins that attenuate the glycemic response. Our experimental data showed that the dissolution time of the films was halved by adding 2% surfactant. The film with the best dissolution properties compared with an increased content of 20 g of bilberry juice and 1.5 g of β-glucan is represented by Sample 7, which dissolves in 50.33 ± 1.52 s.

Moreover, we can conclude that in all of the samples, the water vapor barrier properties are remarkably low. Furthermore, the low water absorption rate (WVTR) between 3.2562 and 7.1111 g × h^−1^ × m^−2^ makes β-glucans/bilberry juice films suitable for packaging dry powdered pharmaceuticals. Ultimately, of course, these values are up to 32 times higher than a plastic film (e.g., LDPE). However, for a fast dissolving bioactive film that is intended for edible packaging, the values are outstanding. Water activity tests and moisture content proved the film stability by values below 0.6 a_w_ and MC up to 14.39%, which will not affect the quality of the packaged products. Since the film-forming solution contains a high amount of β-glucan and bilberry juice (rich in anthocyanidins), a considerably more opaque and reddish film will form after drying. Images of β-glucan/bilberry juice films and laboratory tests had detected the influence of these changes on different levels of significance, while SEM micrographs identified porous and compact structures on the cross section depending on the analyzed samples. FT-IR spectra revealed all of the compounds in film samples. Therefore, no degradation was found for β-glucans and bilberry juice.

The bioactive films developed in the present work could be used for packaging materials along with the bioactive delivering system of releasing compounds in aqueous solutions, which is necessary for people with a special medical condition, such as diabetes. Dispersion of the packaged product will be carried out simultaneously with the dissolution of bioactive film.

## 4. Materials and Methods

### 4.1. Chemicals

Bilberry juice (*Vaccinium myrtillus* L.) was purchased from a local market (distributed by SC. Deco Italia SRL, Suceagu, Cluj, Romania). According to the manufacturer, it is a 100% natural juice from bilberry fruit. The determined dry weight of the clear juice was 11.5% (*w*/*w*) with a pH value of 3.3. β-glucan was extracted from spent brewer’s yeast provided by the SC. Bermas SA. brewery (Suceava, Romania). Other film components were: Sodium alginate, Product No. 9180.1 (Carl Roth, Karlsruhe, Germany), Glycerin, Product No. G7893 (Sigma-Aldrich, ACS reagent ≥ 99.5%, St. Louis, MO, USA), and Soybean oil (oil of genetically unmodified soybeans, Dachim SRL, Cluj, Romania).

#### 4.1.1. β-Glucan Isolation

β-glucan, especially from spent brewer’s yeast, is known to have particular potential in the inducement of innate immune response due to the triple helix structure of the insoluble polysaccharide conformation [46]. The most reliable method used for yeast β-glucan isolation is the alkaline-acid process [47]. Briefly, yeast slurry was purified and debittered at 50 °C with NaOH 2 N (up to pH 10) for 10 min according to [48]. Yeast cells were autolyzed at 55 °C/24 h and then were subjected to an alkaline extraction with NaOH 1.5 N at 90 °C/2 h in a ratio of 1.5 (*w*/*v*) according to [49], followed by an acid treatment with HCl solution for 2 h at 75 °C. The wet extract was washed three times and labeled as yeast β-glucan since it contains this polysaccharide as the principal component [19]. The FT-IR spectra showed characteristic bands for β-1,3 configuration at the wavenumbers of 1153.43 and 1104.87 cm^−1^, while the β-1,6 glucan specific for yeast glucan was found at 889.33 cm^−1^ [43,50].

#### 4.1.2. Film Preparation and Casting

The film-forming solution was prepared from the isolated β-glucan in different proportions and the addition of sodium alginate into each beaker. Therefore, bilberry juice with a dry weight of 11.5% (*w*/*w*) has been measured and prepared for each sample. Glycerin was added as plasticizer as 25% related to the dry weight of the solids of β-glucan, sodium alginate, and bilberry juice. Two percent (*w*/*w*) soybean oil of the total solid weight was added as a surfactant to samples 2, 4, and 7 in order to observe whether there are significant changes between the physical chemical parameters analyzed. Distilled water was added up to a total volume of 150 mL. The mixture was subjected to heating at 80 °C under continuous stirring (900 rpm) for 15 min. After 15 min of stirring in the homogeneous solution, the measured bilberry juice was added in each sample and stirred continuously for another 15 min for incorporation and for slight sterilization (30 min in total).

β-glucan/bilberry juice films were prepared by the casting technique as reported by [51]. Accordingly, equal suspensions from the film-forming solution were poured onto plastic petri dishes and dried at 40 °C for 48 h. The dried films were stored at room temperature prior to the analysis.

### 4.2. Methods

#### 4.2.1. Determination of Thickness

The film thickness was determined with the thickness gauge PosiTector 6000 (DeFelsko, Ogdensburg, NY, USA) with an accuracy of 0.1 µm. Measurements were taken at 10 different points, and the average was used to calculate the film properties. Thickness of the films was expressed in µm.

#### 4.2.2. Determination of Water Vapor Transmission Rate (WVTR)

Water vapor transmission rates were measured using the described standard ASTM E96/96M method [52]. The dry cup method involved sealing films horizontally on a petri dish containing about 10 g CaCl_2_ as desiccant to create 0% RH inside the cups. Samples with desiccant were placed in an environmental chamber with a NaCl solution, which provides 75% RH. The WVTR of the films was calculated by dividing the slope to the area of exposed film using the following equation:

(1) $$WVTR=\frac{∆W}{∆t\times A}\;(g\;\times \;h^{-1}\;\times \;m^{-2})$$

where ∆*W*/∆*t* is the amount of water gained in the unit of time (g/h) and *A* is the area exposed to the water vapor diffusion (m^2^).

#### 4.2.3. Determination of Water Vapor Permeability (WVP)

The permeation characteristic of the β-glucan/bilberry juice films was investigated by dividing the *WVTR* values to the water vapor partial pressure across the film and multiplying by the film thickness (in mm) as described by [53]. The *WVP* was expressed by the following equation:

(2) $$WVP=\frac{WVTR\times L}{∆p}\;(g\;\times \;\mathrm{mm}\;\times \;{\mathrm{kPa}}^{-1}\;\times \;h^{-1}\;\times \;m^{-2})$$

where *WVTR* is the water vapor transmission rate (g × h^−1^ × m^−2^), *L* is the thickness of the film (mm), and ∆*p* is the water vapor partial pressure across the film (kPa) calculated according to the formula:

(3) $$∆p=S\times \left(R_{1}-R_{2}\right)\;(\mathrm{kPa})$$

where *S* is the saturated vapor pressure of water (3.1687 kPa at 25 °C [54]) and the moisture gradients *R*_1_ and *R*_2_ are 0.75 and 0, respectively.

#### 4.2.4. Dissolution Time

The film samples were cut into squares of 2 × 2 cm, immersed in 50 mL of distilled water, and then vigorously shaken until dissolution. The dissolution time (s) was recorded using a chronometer.

#### 4.2.5. Determination of Color and Opacity

Film opacity was determined by measuring the absorbance at 600 nm and dividing by the film thickness [55]. The absorbance was acquired in UV-VIS-NIR Shimadzu 3600 spectrophotometer (Tokyo, Japan). The following equation for *opacity* is shown below:

(4) $$Opacity=Abs_{600nm}/L$$

where $Abs_{600nm}$ is the absorbance (600 nm) and *L* is the film thickness (mm).

The color was quantified according to the CIELab color space (lightness (L), redness (a*), and yellowness (b*)) using a portable chromameter CR-400 (Konica Minolta, Tokyo, Japan).

#### 4.2.6. Water Activity Tests and Moisture Content

The water activity was measured with a water activity analyzer AquaLab 4TE (Meter Group, Inc., Pullman, WA, USA). With the use of chilled-mirror dew point technology, the instrument was able to determine a_w_ values with a resolution of 10^−4^ between 0.03 and 1.

Moisture content was determined gravimetrically according to [56], samples were weighed before and after drying at 105 °C for 24 h, and the difference in weight loss was expressed as the moisture content in films, according to the following equation:

(5) $$MC=\frac{W_{0}-W_{1}}{W_{0}}\times 100\;(\%)$$

where *W*_0_ is the initial weight of the sample and *W*_1_ is the final weight of the dried film.

#### 4.2.7. FT-IR Spectroscopy

FT-IR spectra with attenuated total reflectance unit (ATR) analysis were achieved using Nicolet iS-20 FT-IR spectrometer (Thermo Scientific™, Karlsruhe, Dieselstraße, Germany). Measurements were conducted by placing samples directly on the ZnSe crystal plate. The spectra were collected in the region of 4000–650 cm^−1^ by 32 scans per spectrum at a resolution of 4 cm^−1^.

#### 4.2.8. Scanning Electron Microscopy (SEM)

The cross-section morphology of the films was observed using a scanning electron microscope VEGA II LMU (Tescan, Brno, Czech Republic) under HighVac conditions using a secondary electron (SE) detector operated at an accelerating voltage of 30 kV and a magnification of 1 kx without preliminary coating on the investigated surface.

#### 4.2.9. Dry Weight Determination (*w*/*w*)

Dry weight was determined by weighing about 10 g of juice, solids and dry in an oven at 105 °C to constant weight. Samples were transferred into a desiccator to prevent moisture uptake. The measurements for dry weight were made according to the following equation:

(6) $$DW=\frac{w_{2}-w_{3}}{w_{2}-w_{1}}\times 100\;(\%\;w/w)$$

where *w*_1_ is the weight of crucible; *w*_2_ is the initial weight of crucible with sample, g; and *w*_3_ is the final weight of crucible with sample after drying, g.

#### 4.2.10. Statistical Analysis of the Results

A one-way ANOVA test was used to determine whether there was a statistically significant difference between the means of the independent groups. Table 2 summarized the mean values and standard deviation of the physicochemical test results on a 95% confidence level. All of the tests, except for thickness, which require a mean of minimum 10 data points were expressed as the average and standard deviation of triplicates.

## 5. Conclusions

This study demonstrated good compatibility between yeast β-glucans and bilberry juice in the development of fast dissolving films. The addition of bilberry juice in films is of particular importance in the management of metabolic disorders, such as diabetes. Considering the results, films based on β-glucans/bilberry juice with improved fast dissolving time and good water vapor barrier properties can be a potential novel film for packaging dry powdered pharmaceuticals.

## Figures and Tables

**Table 1:** Composition of β-glucan/bilberry juice blends.

Sample	β-Glucan (BG), g	Sodium Alginate (SA), g	Bilberry Juice (BJ), g	Dry Weight of Bilberry Juice (Determined), g	Total Solids (BG+SA+BJ), g	Glycerin, 25% (*w*/*w*) of the Total Solid Weight, g	Soybean Oil, 2% (*w*/*w*) of the Total Solid Weight, %	Water, Up to Total Volume, mL
1	1	0.8	10	1.15	2.95	0.7375	0	150
2	1	0.8	10	1.15	2.95	0.7375	2	150
3	1	0.8	20	2.3	4.10	1.0250	0	150
4	1	0.8	20	2.3	4.10	1.0250	2	150
5	1.5	0.8	10	1.15	3.45	0.8625	0	150
6	1.5	0.8	20	2.3	4.60	1.1500	0	150
7	1.5	0.8	20	2.3	4.60	1.1500	2	150

**Table 2:** Physicochemical characteristics of β-glucan/bilberry juice film.

Physichochemical Parameters	β-Glucan/Bilberry Juice Film							F-Value
	Sample 1	Sample 2	Sample 3	Sample 4	Sample 5	Sample 6	Sample 7	
Thickness (µm)	66.43 (8.53) ^d^	73.03 (6.03) ^cd^	112.06 (6.66) ^a^	68.96 (9.51) ^d^	83.73 (7.57) ^c^	119.7 (9.39) ^a^	97.8 (6.48) ^b^	22.24 ***
WVTR (g × h^−1^ × m^−2^)	3.7956 (0.2) ^c^	3.6140 (0.16) ^c^	3.2562 (0.35) ^c^	5.9213 (0.48) ^b^	6.8927 (0.68) ^a^	7.1111 (0.62) ^a^	5.9036 (0.56) ^b^	34.4 ***
WVP (g × mm × kPa^−1^ × h^−1^ × m^−2^)	0.1057 (0.009) ^e^	0.1107 (0.004) ^de^	0.1542 (0.02) ^cd^	0.1710 (0.01) ^c^	0.2442 (0.04) ^b^	0.3568 (0.01) ^a^	0.2432 (0.03) ^b^	38.82 ***
L	31.41 (0.34) ^bcd^	30.33 (1.37) ^cd^	29.63 (1.09) ^d^	32.21 (0.80) ^bc^	35.69 (0.58) ^a^	35.5 (1.67) ^a^	32.38 (1.53) ^b^	11.55 ***
a*	1.19 (0.06) ^de^	1.35 (0.07) ^d^	2.82 (0.21) ^a^	2.00 (0.1) ^b^	1.16 (0.02) ^e^	1.75 (0.1) ^c^	2.03 (0.03) ^b^	94.41 ***
b*	1.25 (0.02) ^de^	1.69 (0.11) ^ab^	1.40 (0.18) ^cd^	1.11 (0.07) ^e^	1.53 (0.09) ^bc^	1.53 (0.18) ^bc^	1.79 (0.2) ^a^	8.38 ***
Opacity (Abs × mm^−1^)	0.39 (0.09) ^d^	0.64 (0.007) ^c^	0.85 (0.12) ^bc^	1.11 (0.19) ^a^	1.21 (0.02) ^a^	1.05 (0.04) ^ab^	1.14 (0.21) ^a^	17.38 ***
Dissolution Time (s)	36.66 (1.52) ^e^	22.33 (0.57) ^f^	94 (3.6) ^b^	46.33 (1.52) ^d^	35.33 (2.51) ^e^	105.66 (2.08) ^a^	50.33 (1.52) ^c^	674.08 ***
a_w_	0.3562 (0.004) ^d^	0.3623 (0.003) ^cd^	0.3584 (0.002) ^d^	0.3606 (0.006) ^d^	0.3915 (0.0006) ^a^	0.3690 (0.003) ^c^	0.3765 (0.003) ^b^	28.22 ***
MC (%)	11.14 (0.13) ^d^	10.19 (0.24) ^e^	13.69 (0.29) ^b^	11.16 (0.22) ^d^	14.32 (0.16) ^a^	11.89 (0.12) ^c^	14.39 (0.29) ^a^	181.03 ***

Apart from thickness which is determined in 10 data points (*n* = 10), each value is the mean of three replicates ± standard deviation (*n* = 3). *** Statistically significant at *p* < 0.001. ^a–f^ Different letters in the same rows indicate significant differences between samples. WVTR: Water Vapor Transmission Rate; WVP: Water Vapor Permeability; L, a*, b*: Color profile; a_w_: Water activity; MC: Moisture content.

**Table 3:** The effect of glycerin content on WVTR values.

Sample	Amount of Glycerin, g	WVTR, (g × h^−1^ × m^−2^)
S1, S2	0.7375	3.7048 (0.19) ^b^
S5	0.8625	6.8927 (0.68) ^a^
S3, S4	1.025	4.5887 (1.5) ^b^
S6, S7	1.15	6.5073 (0.84) ^a^
		F-value 12.14 ***

*** Statistically significant at *p* < 0.001. ^a,b^ Different letters in the same column indicate significant differences.

**Table 4:** The influence of β-glucan, soybean oil, and bilberry juice on dissolution time.

Physichochemical Parameter	Composition		
	β-glucan		
	1 g	1.5 g	F-value
Dissolution Time (s)	49.83 (28.14) ^a^	63.77 (32.13) ^a^	1.12 ^ns^
	Soybean oil		
	0%	2%	F-value
Dissolution Time (s)	67.91 (33.68) ^b^	39.66 (13.16) ^a^	5.62 *
	Bilberry juice		
	10 g	20 g	F-value
Dissolution Time (s)	31.44 (7.01) ^b^	74.08 (27.35) ^a^	20.06 ***

^ns^: Not significant; * *p* < 0.05; *** *p* < 0.001. ^a,b^ Different letters in the same rows indicate significant differences.

**Table 5:** The influence of the β-glucan and bilberry juice on opacity and color of the films.

Physichochemical Parameters	Composition		
	β-glucan		
	1 g	1.5 g	F-value
L	30.89 (1.32) ^b^	34.37 (1.91) ^a^	24.25 ***
a*	1.84 (0.67) ^a^	1.64 (0.38) ^a^	0.59 ^ns^
b*	1.36 (0.24) ^b^	1.61 (0.19) ^a^	6.48 **
Opacity (Abs × mm^−1^)	0.74 (0.29) ^b^	1.13 (0.12) ^a^	13.25 ***
	Bilberry juice		
	10 g	20 g	F-value
L	32.48 (2.57) ^a^	32.32 (2.29) ^a^	0.02 ^ns^
a*	1.23 (0.10) ^b^	2.15 (0.43) ^a^	37.99 ***
b*	1.49 (0.2) ^a^	1.46 (0.29) ^a^	0.08 ^ns^
Opacity (Abs × mm^−1^)	0.74 (0.36) ^b^	1.04 (0.17) ^a^	5.77 *

^ns:^ Not significant; * *p* < 0.05; ** *p* < 0.01; *** *p* < 0.001. ^a,b^ Different letters in the same rows indicate significant differences.

## Data Availability

Not applicable.
